# Predicting survival in cardiac arrest patients admitted to intensive care using the Prognosis After Resuscitation score

**DOI:** 10.1186/cc9719

**Published:** 2011-03-11

**Authors:** R Porter, I Goodhart, A Temple

**Affiliations:** 1 Sheffield Teaching Hospitals NHS Trust, Sheffield, UK

## Introduction

Developed from meta-analysis in 1992, the Prognosis After Resuscitation (PAR) score consists of seven, relatively straight-forward to calculate, variables with scores greater than 5 predicting nonsurvival [1]. The aim of this evaluation was to assess PAR scoring as a means of predicting nonsurvival of post-cardiac arrest patients admitted to the general intensive care unit (ITU) at Sheffield Teaching Hospitals NHS Trust (STH).

## Methods

Previous local service reviews have collected data on hospital survival and PAR scoring between January 2002 and May 2008 [2,3]. In addition, from May 2008 to July 2010, post-cardiac arrest patients were identified from the admissions book and a medical notes review was carried out.

## Results

**Figure 1:**
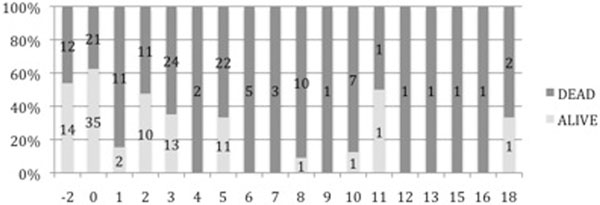
**PAR score and hospital outcome (2002 to 2010)**.

Since 2002 a total of 225 post-cardiac arrest patients have been admitted to the ITU. Forty per cent survived until hospital discharge. The PAR score ranged between -2 and 18, with 0 being the most common score. Four patients from the 37 (13.5%), admitted to the ITU, with a PAR score of greater than 5 survived until hospital discharge. Forty-six per cent of patients with a PAR score of 5 or less survived to hospital discharge. See Figure 1.

## Conclusions

Over the 8 years of review of our data we have only identified four patients where ongoing care was both appropriate and successful despite a PAR score greater than 5. We believe that these patients should have been admitted regardless of the PAR score due to the underlying pathology. The PAR score is an invaluable screening tool in justifying the decision not to admit a patient in whom it is felt critical care is not justified. However, caution must be used as the PAR score should be an aid to clinicians rather than the sole factor deciding appropriateness of critical care admission.
